# Health-related physical fitness and weight status in Hong Kong adolescents

**DOI:** 10.1186/1471-2458-10-88

**Published:** 2010-02-23

**Authors:** Kwok-Kei Mak, Sai-Yin Ho, Wing-Sze Lo, G Neil Thomas, Alison M McManus, Jeffrey R Day, Tai-Hing Lam

**Affiliations:** 1 School of Public Health, University of Hong Kong, Hong Kong; 2 Department of Public Health and Epidemiology, University of Birmingham, Birmingham, UK; 3 Institute of Human Performance, University of Hong Kong, Hong Kong; 4 Faculty of Education, University of Hong Kong, Hong Kong

## Abstract

**Background:**

This study was designed to investigate the relation between health-related physical fitness and weight status in Hong Kong adolescents.

**Methods:**

3,204 students aged 12-18 years participated in the Hong Kong Student Obesity Surveillance (HKSOS) project in 2006-2007. Anthropometric measures (height, weight) and health-related fitness (push-up, sit-up, sit-and-reach, 9-minute run) were assessed. Body mass index (BMI) was computed to classify participants into normal weight, underweight (Grade I, II/III), overweight, and obese groups. The associations of health-related physical fitness with BMI and weight status were examined by partial correlation coefficients and analysis of covariance, respectively.

**Results:**

More boys than girls were overweight or obese (18.0% vs 8.7%), but more girls than boys were underweight (22.3% vs 16.7%). Boys performed significantly (P < 0.001) better in sit-up (38.8 vs 31.6 times/min) and 9-minute run (1632.1 vs 1353.2 m), but poorer in sit-and-reach (27.4 vs 32.2 cm) than girls. All four physical fitness tests were significantly positively correlated with each other in both sexes, and BMI was only weakly correlated with sit up and sit-and-reach tests in boys. Decreasing performance (P for trend < 0.05) was observed from normal weight to overweight and obese for push-up, sit-up, and 9-minute run in both sexes. From normal weight to Grade I and Grade II/III underweight, decreasing performance (P for trend < 0.05) for sit-up and sit-and-reach in both sexes and for push-up in boys was observed.

**Conclusions:**

The relations between BMI and health-related physical fitness in adolescents were non-linear. Overweight/obese and underweight adolescents had poorer performance in push-up and sit-up tests than normal weight adolescents. Different aspects of health-related physical fitness may serve as immediate indicators of potential health risks for underweight and overweight adolescents.

## Background

Overweight in children and adolescents are increasingly common [1] while physical fitness in adolescents is declining [2]. Lower fitness in adolescents may track into adulthood [3]. Previous studies on the effects of adolescent obesity have mainly focused on psychosocial problems [4-6] and typical risk factors of cardiovascular diseases [7,8] while findings on health-related physical fitness are scanty. Health-related physical fitness has the advantage that it can be measured non-invasively, and adolescents would probably find it much easier to relate to being unfit than being high in cholesterol or having chronic diseases in midlife.

The effects of overweight on health-related physical fitness vary with the component of fitness being examined. Compared with normal weight, overweight adolescents tend to have poorer muscular endurance (measured by sit-up) [9], cardiovascular fitness (measured by endurance run) [10,11], but similar flexibility (measured by sit-and-reach) [12,13], and even better isometric strength (measured by handgrip test) [14].

Due to a relatively low prevalence of underweight in Western populations, findings on the health consequences of underweight, including physical fitness, are scarce. In Asia, the desire to be thin is common among young people [15-17]. Many Asian countries, such as China, have the dual burden of both underweight and overweight [18-20]. A recent study revealed that 24% of boys and 15% of girls in Jiangsu province, China were underweight [21]. As a result, investigations on the associations between underweight and physical outcomes such as fitness among adolescents are important. This study aims to examine the relations of health-related physical fitness with BMI and weight status (underweight, normal, overweight/obese) among Chinese adolescents.

## Methods

Anthropometric parameters and health-related physical fitness were assessed in 3,204 Form 1-7 students (equivalent to grade 7 to 12 in the US) aged 12-18 years (50.7% boys) from 4 schools who participated in the Hong Kong Student Obesity Surveillance (HKSOS) project in 2006-2007. Invitation letters were sent to parents for passive consent to participate in the study; only those who declined participation were required to return a signed reply form. Even with parental consent, student participation was totally voluntary. The Institutional Review Board of the University of Hong Kong/Hospital Authority Hong Kong West Cluster has approved the research design and consent procedures.

Height and weight of students were measured barefoot and in light clothing by trained teachers, following the National Health and Nutrition Examination Survey protocol [22]. The school equipment (different electronic scales and wall-mount tapes) was validated against a calibrated Seca stadiometer (Model 844) and Seca electronic scale (Model 214). Body mass index (BMI) was computed [weight (kg)/height squared (m^2^)] to classify participants into overweight and obese groups using the International Obesity Task Force age- and sex-specific BMI cutoffs equivalent to BMI values (kg/m^2^) of 25 and 30, respectively, at age 18 [23]. Similarly, Grade I, II and III underweight were defined using cutoffs equivalent to BMI values of 18.5, 17, and 16, respectively, at age 18 [24]. Due to the relatively small numbers of Grade II and III underweight subjects, they were combined in the analyses.

Four fitness tests were carried out during physical education lessons in schools. They were (i) timed push-up test, (ii) sit-up test, (iii) sit-and-reach test, and (iv) 9-minute distance run. Push-ups and sit-and-reach were used to assess upper body muscular strength and low back flexibility, respectively [25]. Timed sit-ups were carried out to gauge abdominal muscular strength and endurance. Cardiovascular fitness was assessed by a 9-minute distance run in a 15 m × 25 m basketball court. Participants performed the sit-up test with knees bent at 90 degrees and feet flat on the floor. The number of completed sit-ups in 1 minute was recorded. Different methods were used for the push-up tests, with extended legs for boys and bent-knees for girls. In the sit-and-reach test, participants sat on the ground with straight legs against a standard reach box with 23 cm marked at the level of the feet. They were instructed to reach smoothly forward and sustain in the extreme reach position for 2 seconds.

All analyses were performed with stratification by sex. T-tests were performed to examine sex differences in physical fitness. Partial correlation coefficients with adjustment of age were computed to examine the correlations of each fitness test with other fitness tests and BMI. To compare the health-related physical fitness of underweight, overweight and obese adolescents against the normal weight group, analysis of covariance (ANCOVA) with polynomial contrast was conducted for boys and girls separately and with age entered as a covariate.

## Results

Table 1 shows that boys were more likely to be overweight/obese than girls (18.0% vs 8.7%), while girls were more likely to be underweight than boys (22.3% vs 16.7%). Boys performed significantly (P < 0.001) better in sit-up (38.8 vs 31.6 times/min) and 9-minute run (1632.1 vs 1353.2 m), but poorer in sit-and-reach (27.4 vs 32.2 cm) than girls. The mean number of push-ups completed in one minute was 23.2 (SD = 14.6) for boys and 27.9 (SD = 16.3) for girls, but they were not comparable because of the different styles adopted.

**Table 1:** Age distribution, weight status and health-related physical fitness by sex

	Boys (N = 1626)	Girls (N = 1578)	P*
Age (n and %)			
12 or below	288 (17.7)	293 (18.6)	
13	345 (21.2)	297 (18.8)	
14	320 (19.7)	268 (17.0)	
15	264 (16.2)	248 (15.7)	
16	166 (10.2)	212 (13.4)	
17	136 (8.4)	147 (9.3)	
18 or above	107 (6.6)	113 (7.2)	
Weight status (n and %)			
Grade II/III underweight	87 (5.4)	93 (5.9)	
Grade I underweight	183 (11.3)	259 (16.4)	
Normal weight	1064 (65.4)	1089 (69.0)	
Overweight	250 (15.4)	123 (7.8)	
Obese	42 (2.6)	14 (0.9)	
Physical fitness tests (mean and SD)			
Push-up (count/min)	23.2 (14.6)	27.9 (16.3)	N/A
Sit-up (count/min)	38.8 (10.2)	31.6 (8.4)	< 0.001
Sit-and-reach (cm)	27.4 (8.7)	32.3 (8.0)	< 0.001
9-min run (m)	1632.1 (299.5)	1353.2 (211.1)	< 0.001

* P-value for sex difference based on t-test

Table 2 shows that all fitness test items were significantly positively correlated with each other with coefficients ranging from 0.15 to 0.45 in boys and from 0.18 to 0.41 in girls (all P < 0.001). The associations between BMI and fitness test items differed by sex. In boys, BMI correlated only weakly with sit-up test (r = 0.05, P < 0.05) and sit-and-reach test (r = 0.06, P < 0.05). In girls, no significant correlation between BMI and fitness test was found.

**Table 2:** Partial correlation^a ^between BMI and fitness tests in boys and girls

	**BMI**	**Push-up**	**Sit-up**	**Sit-and-reach**	**9-min run**
	
Boys					
BMI		0.16	0.05*	0.06*	-0.007
Push-up			0.45**	0.22**	0.35**
Sit-up				0.23**	0.41**
Sit-and-reach					0.15**
9-min run					
Girls					
BMI		-0.16	-0.01	-0.001	-0.05
Push-up			0.41**	0.26**	0.31**
Sit-up				0.21**	0.40**
Sit-and-reach					0.18**
9-min run					

^a^Adjusted for age

* P < 0.05

** P < 0.001

Additional file 1, Table S3 shows that after controlling for age, the push-up, sit-up, and 9-minute run performance in boys and girls declined from normal weight to overweight and obese groups (P for trend < 0.05). Similar decreasing performance from normal weight to Grade I and Grade II/III underweight was observed for sit-up and sit-and-reach in both sexes and for push-up in boys (all P for trend < 0.05).

## Discussion

The prevalence of underweight in our study (boys: 16.7%; girls: 22.3%) are higher than that in Japan (about 3% in both sexes) [26], Portugal (boys: 3.9%; girls 5.6%) [27] and Turkey (boys: 14.4%; girls: 11.1%) [28]. With such a substantial prevalence of both underweight and overweight/obesity in the sample, we found that normal weight Hong Kong adolescents generally had better physical fitness than their underweight and overweight counterparts. Such an association between weight status and health-related physical fitness has also been observed and was described as an inverted J-shape association by Bovet et al. [29]. Our findings support that the relation between BMI and health-related physical fitness is non-linear [30], especially when the full spectrum of weight status from underweight to obesity is considered. Boys achieved better results than girls in muscular strength (push-up, sit-up) and cardiovascular fitness (9-minute run) tests. Consistent with the literature [31,32], girls performed better than boys in the sit-and-reach test on flexibility.

### Overweight and obesity

Overweight and obese boys and girls both performed poorer in push-up, sit-up and endurance running compared with normal weight, which is consistent with previous studies [9,10,33]. However, such differences need to be interpreted cautiously due to the higher energy cost of lifting a greater body mass by overweight subjects [9,34]. Artero et al. have shown that the deficit in weight-bearing fitness tests of overweight and obese adolescents was either attenuated or even reversed after adjusting for fat mass [14]. Similarly, cardiorespiratory fitness measured by VO_2 _max did not differ between obese and normal weight adolescents, after adjusting for body composition [35]. Therefore, non-weight bearing tests may better reflect the physical fitness of adolescents with different weight status.

As regards flexibility, we found that overweight/obese and normal weight adolescents had similar sit-and-reach results. This is in line with two Taiwanese studies [12,13] but in contrast to a Western report that slightly better sit-and-reach results were achieved by overweight than normal weight girls [36].

### Underweight

We found a significant decreasing trend of performance in sit-and-reach and sit-up tests from normal weight to Grade I and Grade II/III underweight in both boys and girls. Poorer sit-and-reach results in underweight boys and girls [14,36], as well as null findings [12], have been reported by others. It is unclear why underweight adolescents have poorer sit-and-reach performance which mainly reflects hamstring and low back flexibility. Confirmation of this finding by other studies is needed. As underweight is associated with poorer sit-up test in the present study and a previous report [29], it would be interesting to investigate if weaker abdominal strength may affect the ability of underweight subjects to flex and sustain the trunk in the extreme reach position. It should be noted that although greater flexibility is generally believed to be beneficial to health, current evidence is inconclusive [37].

Prista et al. found that underweight adolescents had poorer running endurance than the normal weight group [36]. In another study, lean girls were found to score higher in relative maximal power output (per unit body size) than the normal group [38]. In our sample, there was an insignificant trend (P = 0.096) of lower 9-minute run results from normal weight to Grade I and Grade II/III underweight in boys but no clear trend was observed in girls. While underweight adolescents may have weaker lower limb muscles, they have the advantage of a lighter weight in endurance running. Both underweight boys and girls were reported to have poorer push-up performance compared with normal weight [29], but this was observed only in boys in the present study.

### Limitations and strengths

This study has several limitations. First, the sample may not truly represent the entire adolescent population in Hong Kong as it was not randomly chosen. Nonetheless, it is probably sufficient for demonstrating the weight status differences in physical fitness. Second, the physical fitness results may not truly reflect actual physical functioning, and variations across fitness batteries exist. Most of our fitness items required different levels of body lifting, which disadvantaged heavier subjects. No test-retest was conducted to determine the reliability of our fitness tests, but high reliability of similar tests among adolescents has been reported by others [39]. Furthermore, the fitness tests were conducted as a component of continuous assessment of school performance under the supervision of the well-trained teachers, it is reasonable to believe that students performed the tests with similar effort and in a similar manner.

Third, the lack of an additional indicator of body composition, such as percentage body fat, has made it impossible to adjust for differences in fat mass [35]. Fourth, pubertal stages of the adolescents were not assessed. While boys who are early maturers may perform better in physical fitness tests than their peers due to muscle development, early maturing girls may perform poorer for their relatively higher fat mass [40]. Although physical examination of Tanner staging is difficult, self-reported sexual maturity questionnaires can be used as a valid alternative in future studies [41,42].

Hong Kong has provided unique data because of the coexistence of substantial proportions of underweight and overweight adolescents. This permitted us to examine trends of findings across a wide spectrum of weight status. Body dissatisfaction and unhealthy weight-control behaviours are common among girls who want to be thinner [43], our findings can be used to warn them of the potential harm of underweight on physical fitness.

To remedy the fitness deficits in underweight and overweight adolescents, promoting leisure physical activities rather than regimented training may have less destructive effects on their sense of physical self [44]. Perhaps, as a first step, health implications of both lower fitness and abnormal weight status could be emphasized in the physical education curriculum to raise the awareness of students [45]. Further studies examining the tracking of the association between weight status and physical fitness from adolescence to adulthood are warranted.

## Conclusions

The relation between BMI and health-related physical fitness was non-linear in Hong Kong adolescents. Overweight/obese and underweight boys and girls had poorer performance in push-up and sit-up tests than their normal weight counterparts. Overweight/obese boys and girls and underweight boys were also poorer in the 9-minute run test. Underweight but not overweight/obese boys and girls performed poorer in the sit-and-reach test compared with normal weight. Different aspects of health-related physical fitness may serve as immediate indicators of potential health risks for both underweight and overweight youngsters.

## Competing interests

The authors declare that they have no competing interests.

## Authors' contributions

KKM contributed to study design and management, performed statistical analyses and drafted the manuscript; SYH is the principle investigator of the HKSOS project and critically revised the manuscript; WSL contributed to coordination of the study and revision of the manuscript; GNT, AMM, and JRD made important revisions to the manuscript. THL oversaw the development of the project and critically revised the manuscript. All authors read and approved the final manuscript.

## Pre-publication history

The pre-publication history for this paper can be accessed here:

http://www.biomedcentral.com/1471-2458/10/88/prepub

## Supplementary Material

Additional file 1 **Table S3**. Analysis of covariance of physical fitness tests by weight status. Click here for file
