# Supplementary material for: Health-related physical fitness and weight status in Hong Kong adolescents
Source: BMC Public Health. 2010 Feb 23;10:88. doi: 10.1186/1471-2458-10-88 (PMC2836297; doi:10.1186/1471-2458-10-88)
Supplement: Additional file 1 — Table S3. Analysis of covariance of physical fitness tests by weight status. [file 1471-2458-10-88-S1.DOC]

Table S3: Analysis of covariance^a^ of physical fitness tests by weight status

|  | Normal  weight | Grade I underweight | Grade II/III underweight |  |  | Overweight | Obese |  |
| --- | --- | --- | --- | --- | --- | --- | --- | --- |
|  | Mean (SE) | Mean (SE) | Mean (SE) | P for trend* |  | Mean (SE) | Mean (SE) | P for trend** |
| Boys |  |  |  |  |  |  |  |  |
| Push-up  (count/min) | 24.5 (0.5) | 23.6 (1.1) | 21.5 (1.6) | 0.048 |  | 18.8 (0.9) | 15.7 (2.2) | <0.001 |
| Sit-up  (count/min) | 39.8 (0.3) | 38.3 (0.8) | 35.3 (1.1) | <0.001 |  | 36.8 (0.6) | 35.9 (1.6) | 0.021 |
| Sit-and-reach  (cm) | 27.8 (0.3) | 26.2 (0.6) | 24.3 (0.9) | <0.001 |  | 27.8 (0.6) | 27.3 (1.3) | 0.79 |
| 9-min run  (m) | 1664.7 (9.3) | 1659.1 (21.1) | 1616.4 (30.7) | 0.096 |  | 1503.4 (18.3) | 1422.5 (44.7) | <0.001 |
| Girls |  |  |  |  |  |  |  |  |
| Push-up  (count/min) | 27.9 (0.5) | 29.4 (1.0) | 30.4 (1.7) | 0.17 |  | 23.9 (1.4) | 17.3 (4.2) | 0.013 |
| Sit-up  (count/min) | 31.9 (0.3) | 31.7 (0.5) | 30.0 (0.9) | 0.033 |  | 30.1 (0.8) | 22.4 (2.2) | <0.001 |
| Sit-and-reach  (cm) | 32.4 (0.2) | 32.0 (0.5) | 30.2 (0.8) | 0.005 |  | 32.6 (0.7) | 31.0 (2.2) | 0.53 |
| 9-min run  (m) | 1358.5 (6.2) | 1382.1 (12.9) | 1371.9 (21.5) | 0.60 |  | 1242.9 (18.4) | 1140.0 (53.7) | <0.001 |

^a^ Adjusted for age

*P for trend (from normal weight to Grade I underweight and Grade II/III underweight)

** P for trend (from normal weight to overweight and obese)
